# Technical evaluation of TomoTherapy automatic roll correction

**DOI:** 10.1120/jacmp.v16i3.4836

**Published:** 2015-05-08

**Authors:** Steve Laub, Michael Snyder, Jay Burmeister

**Affiliations:** ^1^ Department of Medical Physics CDH Proton Center Warrenville IL; ^2^ Department of Oncology Wayne State University, Karmanos Cancer Center Detroit MI USA

**Keywords:** TomoTherapy, IGRT, roll correction, patient positioning, correction vector

## Abstract

The TomoTherapy Hi·Art System allows the application of rotational corrections as a part of the pretreatment image guidance process. This study outlines a custom method to perform an end‐to‐end evaluation of the TomoTherapy Hi·Art roll correction feature. A roll‐sensitive plan was designed and delivered to a cylindrical solid water phantom to test the accuracy of roll corrections, as well as the ability of the automatic registration feature to detect induced roll. Cylindrical target structures containing coaxial inner avoidance structures were placed adjacent to the plane bisecting the phantom and 7 cm laterally off central axis. The phantom was positioned at isocenter with the target‐plane parallel to the couch surface. Varying degrees of phantom roll were induced and dose to the targets and inner avoidance structures was measured using Kodak EDR2 films placed in the target‐plane. Normalized point doses were compared with baseline (no roll) data to determine the sensitivity of the test and the effectiveness of the roll correction feature. Gamma analysis comparing baseline, roll‐corrected, and uncorrected films was performed using film analysis software. MVCT images were acquired prior to plan delivery. Measured roll was compared with induced roll to evaluate the automatic registration feature's ability to detect rotational misalignment. Rotations beyond 0.3° result in statistically significant deviation from baseline point measurements. Gamma pass rates begin to drop below 90% at approximately 0.5° induced rotation at 3%/3 mm and between 0.2° and 0.3° for 2%/2 mm. With roll correction applied, point dose measurements for all rotations are indistinguishable from baseline, and gamma pass rates exceed 96% when using 3% and 3 mm as evaluation criteria. Measured roll via the automatic registration algorithm agrees with induced rotation to within the test sensitivity for nearly all imaging settings. The TomoTherapy automatic registration system accurately detects induced rotations, and the method presented here for evaluation of the roll correction feature is easily implemented by any clinic with a TomoTherapy Hi·Art unit. This method is sensitive to well within half a degree and demonstrates that the TomoTherapy Hi·Art roll correction feature accurately corrects for induced rotational misalignments to within this level of uncertainty.

PACS numbers: 87.53.Jw, 87.53.Kn, 87.55.Qr, 87.57.nj

## I. INTRODUCTION

Highly conformal radiotherapy treatments necessitate a high degree of setup certainty in order to accurately target the tumor while sparing healthy tissue. As past studies have shown, misalignments in intensity‐modulated radiation therapy (IMRT) treatments are not uncommon and can result in clinically relevant dose delivery errors.^1^, ^2^ The TomoTherapy Hi·Art (Accuray Inc., Sunnyvale, CA) treatment unit utilizes a megavoltage computed tomography (MVCT) scan before each treatment fraction to ensure proper alignment. This MVCT is registered to the planning CT and a correction vector is calculated for the patient's position. The registration process allows for six degrees of freedom: three translations, and three rotations — pitch, yaw, and roll. Since this unit treats patients in a helical fashion, corrections for roll are relatively easily implemented by rotating the delivery pattern about the central axis of the treatment unit such that the dose distribution is rotated by the same amount as the patient. A roll correction feature is provided within the Hi·Art delivery system. However, there are currently no manufacturer recommended tests or published studies devoted to end‐to‐end evaluation of the TomoTherapy Hi·Art roll correction system. In this study, we intend to achieve three goals: design a test sensitive to small rotations that can be used to perform an end‐to‐end evaluation of TomoTherapy's roll correction feature, evaluate TomoTherapy's ability to detect roll, and verify TomoTherapy's ability to correct for rotational misalignment.

## II. MATERIALS AND METHODS

**Figure 1:**
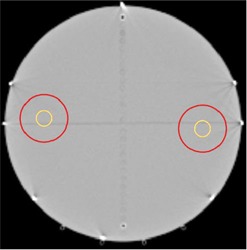
Structure set designed to detect rotational misalignment using Kodak EDR2 film measurements. Red circles denote target stuctures. Yellow circles are designated avoidance regions. Structures are offset from the bisecting plane to maximize baseline sensitivity by placing the highest gradient on the bisecting plane.

A treatment plan was designed for delivery on the TomoTherapy cylindrical “cheese” phantom that would provide a very high gradient region in the film plane, thus making the measured dose distribution in this plane most sensitive to rotations in the “roll” direction. A cylindrical structure measuring 6 cm in diameter and 5 cm in length, designated as the target, was positioned 10 cm from the phantom's central axis in the patient–right direction and 0.7 cm anterior to the bisecting film plane. Another structure of the same length and diameter, also designated as a target, was positioned 10 cm from the phantom's central axis in the patient–left direction and 0.7 cm posterior to the bisecting film plane. Two structures designated as avoidance regions, measuring 2 cm in diameter and 5 cm in length, were positioned coaxially within the target structures. This geometry results in the centers of the structures receiving the lowest dose, with progressively more dose delivered towards the periphery of the avoidance regions, and full dose being delivered to the target structures. The dose gradient inside the avoidance region was designed to be as steep as possible to provide the highest sensitivity to phantom roll. Figure 1 shows the designed structure set. The phantom was placed at the central axis of the treatment unit and the structures were placed as far from the central axis as possible to maximize their movement with induced roll. However, care was also taken to assure that they are deep with respect to the buildup region to allow accurate dose calculation within the structures. A treatment plan was developed and optimized for these structures in the same manner as any clinical treatment plan, with the primary goal being the maximization of the gradient across the film plane.

Our goal was to create a test using the standard phantom and standard phantom geometry used for TomoTherapy patient‐specific delivery quality assurance, thus making it easy for others to repeat this test on their own treatment units. The treatment plan was designed to place the film plane in a region of very high‐dose gradient, thus making it most sensitive to small rotations of the phantom. We were able to achieve a dose gradient of roughly 15% per millimeter, as measured in the planning system. To obtain this gradient, we allowed the maximum dose to the PTV to be 120% of the prescribed dose, with a minimum dose of 75%. We put a maximum dose restriction on the OAR structure of 75%, with a DVH restriction of no more than 55% of the volume receiving 50% of the prescription dose. In addition, the symmetry of the structures provides a redundancy check, as a phantom rotation will induce an identical change in delivered dose to the film areas corresponding to either structure. Film measurements were made using Kodak EDR2 film (Kodak, Rochester, NY).

Six films were irradiated with the phantom at 0° rotation. These measurements, taken at the beginning and end of each measurement session, served both as a baseline data set for comparison with the test data, and as a means to monitor output variations between measurement sessions, which were found to be negligible.

Test films were irradiated with the phantom rotated 0.1°, 0.2°, 0.3°, 0.5°, 1.0°, 2.0°, 3.0°, and 5.0° clockwise (assuming the phantom is viewed from the foot of the couch) and −0.5°, −1.0°, and −3.0° counterclockwise. The phantom was positioned at the virtual isocenter, using the room lasers for translational positioning, and a Mitutoyo (Kawasaki, Japan) Digital Protractor Pro 360 digital level, accurate to 0.1° between ±10°, was used for rotational positioning. Rotation was verified inside the bore after the couch shifted into the bore.

Two film measurements of the treatment delivery were made at each angle, one with roll correction applied and one without. The measurements made without roll correction will illustrate the effects of the induced rotational misalignment on the delivered dose distribution. We intend to establish a threshold at which these misalignments result in a statistically significant change in the delivered dose. The measurements made with roll correction applied will show how well the TomoTherapy roll correction feature corrects for the induced rotational misalignment through comparison with the baseline dose distribution.

Prior to irradiation, a full MVCT scan of the phantom was acquired at each angle. The automatic registration feature was then used to calculate the rotational misalignment relative to the planning CT. These measurements are intended to quantify the ability of the automatic registration feature to detect roll. Measurements were made using the Bone, Bone + Tissue, and Full Image settings. A combination of high‐density and low‐density plugs was inserted into the cheese phantom to provide a reference for registration.

After irradiation, all films were processed with a Kodak M6‐B film processor. Once processed, films were digitized using a VXR‐16 DosimetryPRO Vidar film scanner (Vidar Systems Corp., Herndon, VA) and analyzed using RIT113 software (Radiological Imaging Technology, Colorado Springs, CO).

**Figure 2:**
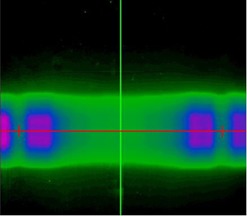
Dose distribution measured with film. The central axis serves as a normalization point, left and right crosses represent test points. High‐dose regions on the left and right side of the film contain the gradient where the test points are located.

Three points of interest were considered in each film: the central point in the film dose distribution and the left and right dose minima. The left and right minima, considered independently, are the test points. The central point in the dose distribution is used as a normalization point. Since the phantom is aligned to the center of the TomoTherapy bore, this point falls on the phantom's central axis and the delivered dose is independent of phantom rotation. The ratio of the minima to the normalization point in each film was used for analysis, in preference to absolute values, to account for any potential variability in the TomoTherapy unit's output and/or in film response.^3^, ^4^, ^5^ The described points are illustrated in Fig. 2, which shows an example film distribution. The central axis serves as the normalization point; the crosses on either side of the film represent the locations of the test points. The specific test points used in each film were found by creating a horizontal line profile across the center of the dose distribution and finding the minimum dose value for the left and right aspects of the distribution.

To determine whether the test points are statistically distinct from the baseline readings, Gaussian distributions were constructed using baseline measurements, again considering the left and right structures independently. Both uncorrected and roll‐corrected data were then compared to these distributions. Test points falling within 3 SD of the baseline average are considered to be statistically similar to the baseline data points. Points falling outside 3 SD of the baseline average are not considered to be a part of the baseline dataset.

Comparing uncorrected data with the baseline Gaussian distribution shows the minimum detectable rotational misalignment, which establishes the sensitivity of the test. The same analysis of the roll‐corrected data demonstrates the TomoTherapy unit's ability to correct for rotational misalignment and reproduce baseline readings.

For further statistical analysis, the data were tested for normality and a *t*‐test was used to compare corrected data with baseline data.^(6)^ Based on the average standard deviation and sample of size of each population, a test statistic was calculated to compare the null hypothesis to an alternative hypothesis. The null hypothesis is that the mean values of the corrected dataset and the baseline dataset are equal. The test statistic is then compared to a rejection region, which is determined by the degrees of freedom and level of the test. If the test statistic falls within the rejection region, the null hypothesis is rejected at that level. This test was used to further statistically confirm whether the TomoTherapy unit is able to accurately correct for roll. There should be no significant differences between the baseline dataset and the corrected data if TomoTherapy's roll correction feature accurately accounts for rotational misalignments.

Finally, gamma analysis was used to compare test films with a baseline film, using both 3% dose difference and 3 mm distance to agreement and 2%/2 mm as acceptance criteria.^(7)^ This test mimics typical patient‐specific delivery quality assurance tests, and is a redundant check of the sensitivity of this test procedure to rotations and the ability of the roll correction feature to correct for these rotations within the detection capability of this gamma analysis.

## III. RESULTS & DISCUSSION

The results for the automatic registration evaluation are presented first, followed by the evaluation of the dose distributions with and without induced roll and the discussion of the results of the roll correction evaluation. 1, 2 show the results of the automatic registration calculation performed before each test measurement. The data are organized to show the angle of rotation and the result from each scan setting.

**Table 1:** Baseline roll calculated using TomoTherapy's automatic registration.

*Film*	*Bone (°)*	Bone+Tissue (°)	*Full Image (°)*
1	0.3	0.3	0.3
2	0.3	0.3	0.2
3	0.3	0.3	0.1
4	0.3	0.2	0.2
5	0.4	0.2	0.2
6	0.3	0.2	0.2
Average	0.32	0.25	0.2

**Table 2:** Induced roll and roll calculated using TomoTherapy's automatic registration at each tested angle.

*Induced Roll (°)*	*Bone*	Bone+Tissue	*Full Image*
0.1	0.2	0.2	0.2
0.2	0.5	0.6	0.2
0.3	0.5	0.4	0.3
0.5	0.6	0.6	0.7
1.0	1.3	1.3	1.3
2.0	2.2	2.3	2.3
3.0	2.7	2.7	2.7
5.0	5.3	5.3	5.3
−0.5	0.0	0.0	0.0
−1.0	−1.1	−0.5	−0.7
−3.0	−2.9	−3.0	−3.1

### A. Automatic registration

Table 1 shows an average baseline rotation of approximately 0.26°. This offset is found systematically across multiple measurement sessions and is observed in the induced roll measurements, as well. It could potentially be due to phantom rotation or couch roll at the time of the initial CT scan. However, since this offset cannot be definitively explained, no correction to the data is made. Table 3 shows the difference between roll calculated using TomoTherapy's automatic registration feature and the induced roll. The average difference between the calculated and induced roll is 0.23°, 0.26°, and 0.22° for each of the imaging settings: Bone, Bone + Tissue, and Full Image, respectively. While there appears to be a systematic error in the relative rotation of the phantom between the simulation kVCT and the TomoTherapy MVCT, TomoTherapy's automatic registration feature is able to calculate roll to within the sensitivity of the test for nearly all imaging settings.

**Table 3:** Differences between induced roll and roll calculated using TomoTherapy's automatic registration at each tested angle.

*Induced Roll (°)*	$Bone\;(\delta^{\circ })$	$Bone+Tissue\;(\delta^{\circ })$	$Full\;Image\;(\delta^{\circ })$
0.1	0.1	0.1	0.1
0.2	0.3	0.4	0.0
0.3	0.2	0.1	0.0
0.5	0.1	0.1	0.2
1.0	0.3	0.3	0.3
2.0	0.2	0.3	0.3
3.0	0.3	0.3	0.3
5.0	0.3	0.3	0.3
−0.5	0.5	0.5	0.5
−1.0	0.1	0.5	0.3
−3.0	0.1	0.0	0.1
Average	0.23	0.26	.22

### B. Automatic roll correction

4, 5, 6 show the film measurement results. The data are organized to show the angle of rotation of each film, the normalization value in that film, the test points recorded as absolute dose for the left and right side of each film, and the ratio of the test points to the normalization value. Table 4 shows the six baseline measurements. As none of the baseline measurements were made consecutively, the standard deviation implies that the test setup is highly reproducible. Table 5 shows the measurements from films irradiated with roll correction applied. It is worth noting that the standard deviations of the corrected data match the corresponding standard deviations of the baseline data. While not conclusive, this similarity is expected if the roll correction feature accurately accounts for the induced rotations. Table 6 shows our measurements made with no correction applied. The average and standard deviation of these test points were not recorded as they have no significance to this test.

**Table 4:** Baseline films: normalization values, measurements, and ratios for left (L) and right (R) test points.

*Film*	*Norm*	*L (cGy)*	*R (cGy)*	*L/Norm*	*R/Norm*
1	54.33	113.06	109.17	2.08	2.01
2	53.46	110.90	111.88	2.07	2.09
3	53.97	114.08	115.09	2.11	2.13
4	54.10	109.58	112.83	2.03	2.09
5	54.92	116.45	114.10	2.12	2.08
6	55.46	118.21	114.59	2.13	2.07
Average	54.37	113.71	112.94	2.09	2.08
SD	0.71	3.26	2.19	0.04	0.04

**Table 5:** Normalization values, measurements, and ratios for films irradiated with roll correction applied.

*Angle (°)*	*Norm*	*L (cGy)*	*R (cGy)*	*L/Norm*	*R/Norm*
*0.1*	*53.95*	*114.35*	*111.44*	*2.12*	*2.07*
0.2	53.42	116.21	112.20	2.18	2.10
0.3	53.81	114.53	114.66	2.13	2.13
0.5	53.48	111.13	112.52	2.08	2.10
1.0	54.31	113.78	113.02	2.10	2.08
2.0	54.47	116.03	115.21	2.13	2.12
3.0	54.48	110.53	113.33	2.03	2.08
5.0	55.21	114.13	110.44	2.07	2.00
−0.5	54.97	117.23	114.32	2.13	2.08
−1.0	55.14	118.08	112.58	2.14	2.04
−3.0	55.15	116.61	116.02	2.11	2.10
Average	54.40	114.78	113.25	2.11	2.08
SD	0.67	2.39	1.67	0.04	0.04

**Table 6:** Normalization values, measurements, and ratios for films irradiated without roll correction applied.

*Angle (°)*	*Norm*	*L (cGy)*	*R (cGy)*	*L/Norm*	*R/Norm*
0.1	53.16	113.39	109.88	2.13	2.07
0.2	53.32	108.92	106.44	2.04	2.00
0.3	53.93	107.83	105.07	2.00	1.95
0.5	53.31	96.24	97.27	1.81	1.82
1.0	53.72	84.15	83.97	1.57	1.56
2.0	53.75	65.48	65.57	1.22	1.22
3.0	54.61	54.14	54.11	0.99	0.99
5.0	54.61	58.23	59.61	1.07	1.09
−0.5	53.73	123.88	124.12	2.31	2.31
−1.0	54.49	139.59	141.46	2.56	2.60
−3.0	54.83	193.79	190.20	3.53	3.47
Average	53.95	–	–	–	–
SD	0.59	–	–	–	–

Table 7 shows the difference between the left/right test points and their respective baseline averages for both roll‐corrected and uncorrected data. The left‐side baseline average is 2.09; the right‐side average is 2.08. The standard deviation for both sides is 0.04. The test points that fall further than 3 SD from the respective baseline average are noted.

**Table 7:** Deviation of left and right test points from the respective baseline average. ${\text{Numbers}}^{a}$ fall outside 3 SD from the baseline value.

*Uncorrected*			*Corrected*		
*Angle (°)*	*Left*	*Right*	*Angle (°)*	*Left*	*Right*
0.1	0.04	−0.01	0.1	0.03	−0.01
0.2	−0.05	−0.08	0.2	0.08	0.02
0.3	−0.09	−0.13^a^	0.3	0.04	0.05
0.5	−0.29^a^	−0.25^a^	0.5	−0.01	0.03
1.0	−0.52^a^	−0.51^a^	1.0	0.00	0.00
2.0	−0.87^a^	−0.86^a^	2.0	0.04	0.04
3.0	−1.10^a^	−1.09^a^	3.0	−0.06	0.00
5.0	−1.02^a^	−0.99^a^	5.0	−0.02	−0.08
−0.5	0.21^a^	0.23^a^	−0.5	0.04	0.00
−1.0	0.47^a^	0.52^a^	−1.0	0.05	−0.04
−3.0	1.44^a^	1.39^a^	−3.0	0.02	0.03
*Baseline*					
	*Left*	*Right*			
Average	2.09	2.08			
SD	0.04	0.04			

In the uncorrected dataset, left‐side data points at 0.5° and greater differ from the baseline value by more than 3 SD. Right‐side data exceed 3 SD, beginning at 0.3°. All other angular deviations resulted in measurements that are statistically distinct from the baseline dataset. Using 3 SD as acceptance criteria, we can say that our test is able to detect rotational misalignments greater than or equal to 0.3°.

In contrast to the uncorrected data, the majority of the corrected data points fall within 1 SD of the baseline average and all corrected points fall within 2 SD. Again using 3 SD as acceptance criteria, we can conclude that the roll correction feature was able to accurately modify dose delivery for all tested rotational misalignments to within the accuracy of this test.

The *t*‐test was performed at the α=0.05 (95% confidence) level to compare our corrected data to our baseline dataset. Our test statistic was calculated to be 0.9 and 0.3 for the left and right side, respectively. The datasets corresponding to the left and right sides both have 15 degrees of freedom, which results in a rejection region greater than 2.1. For both the left and right sides, the test statistic falls well short of the rejection region at this level. The data, therefore, show no evidence that the two sets are statistically distinct at this level. The results of these tests strongly suggest that the roll correction feature has accurately corrected the dose delivery to account for the induced rotations.

Gamma analyses were performed comparing the test films to a baseline film. Table 8 shows the percent gamma pass rate of the left and right side data using 3%/3 mm as acceptance criteria.

**Table 8:** 3%/3 mm gamma pass rates. ${\text{Numbers}}^{a}$ are below 90% pass rate commonly used for clinical measurements.

*Angle (°)*	*Roll Corrected*		*Uncorrected*	
	*Left Side*	*Right Side*	*Left Side*	*Right Side*
0.1	100.0	100.0	99.9	100.0
0.2	100.0	100.0	100.0	99.8
0.3	98.4	99.3	95.2	92.3
0.5	99.6	99.7	81.4^a^	78.5^a^
1.0	99.9	100.0	72.3^a^	62.9^a^
2.0	96.8	97.1	54.9^a^	47.3^a^
3.0	99.9	99.4	44.7^a^	42.6^a^
5.0	99.8	99.4	59.4^a^	56.5^a^
−0.5	99.9	99.0	92.5	95.2
−1.0	96.4	99.8	77.0^a^	70.1^a^
−3.0	99.9	99.9	50.8^a^	49.2^a^

Numbers below the 90% pass rate commonly used for clinical patient‐specific quality assurance measurements are noted. The uncorrected data points begin to fall below this threshold at 0.5° rotation. All subsequent positive rotations fall below 90%. The only negative rotation to produce a gamma pass rate above 90% was −0.5°; all other pass rates were below this tolerance. All gamma pass rates corresponding to corrected data points exceed 96%, with an average of 99.1% for the left side and 99.4% for the right.

Uncorrected rotations show a clear trend in decreasing gamma pass rate. All rotations greater than 0.3° would result in films that would be considered clinically unacceptable by these criteria if they were measured for patient‐specific quality assurance, with the counterclockwise rotation of −0.5° being the only exception. However, this is likely the result of the region of interest (ROI) used for the measurement. If a larger ROI was used that encompassed both aspects of the distribution, it is likely that the gamma pass rate at this angle would fall below 90%. After applying roll correction, all failing points were brought up to a passing level near 100%. As we found with our previous statistical tests, this suggests that TomoTherapy's roll correction feature accurately corrects for our induced rotations.

Gamma analysis was also performed using 2%/2 mm pass criteria, the results of which are shown in Table 9. We typically use 3%/3 mm criteria for our clinical patient‐specific QA measurements, as is used in TG‐119.^(8)^ However, 2%/2 mm provides a more sensitive test with which to test these films. Most of the corrected films result in pass rates greater than 90%, even at 2%/2 mm, but some are slightly lower.

**Table 9:** 2%/2 mm gamma pass rates. ${\text{Numbers}}^{a}$ are below 87.6% pass rate, the composite confidence limit stated in TG‐119.

*Angle (°)*	*Roll Corrected*		*Uncorrected*	
	*Left Side*	*Right Side*	*Left Side*	*Right Side*
0.1	100.0	100.0	99.4	99.1
0.2	91.9	100.0	100.0	92.9
0.3	96.6	98.0	83.4^a^	81.6^a^
0.5	98.0	99.2	75.3^a^	71.1^a^
1.0	99.5	99.9	66.4^a^	52.2^a^
2.0	89.7	95.9	45.2^a^	39.9^a^
3.0	98.8	98.8	33.0^a^	37.8^a^
5.0	91.6	98.1	46.0^a^	47.4^a^
−0.5	88.6	96.6	66.4^a^	80.5^a^
−1.0	89.1	95.9	54.3^a^	49.6^a^
−3.0	94.2	95.8	25.7^a^	38.7^a^

TG‐119 reports a composite film confidence limit of 87.6% when using 3%/3 mm gamma pass criteria. Our uncorrected data begin to fall below this limit at 0.3° induced rotation, with no passing points at negative rotations. It is clearly observed that the uncorrected results drop very quickly below the corrected data past 0.2°. The corrected data all exceed the TG‐119 confidence limit even at 2%/2 mm, with an average pass rate of 96.2%. The favorable passing rates, even at 2%/2 mm, indicate a robust testing method, and demonstrate the ability of the roll correction feature to accurately correct for induced rotations.

To put this test sensitivity in context, TG‐148 recommends a tolerance of 1° for gantry angle accuracy and consistency.^(9)^ In the experience of the authors, field service engineers will adjust the position of the gantry if it falls outside of a 0.2° tolerance. With respect to patient treatment, a 0.2° roll would result in a rotational movement of approximately 0.2 mm on the surface of a patient's head or slightly less than 1 mm on the surface of the pelvis, abdomen, or thorax of a very large patient. Such movements are within the strictest recommended tolerances for patient alignment. Furthermore, most targets and OARs are closer to the center of the patient where alignment uncertainty due to roll is even smaller. Thus the sensitivity of this test is adequate to assure accuracy of roll corrections to within clinically relevant tolerances. It should be noted that the 3%/3 mm pass criteria was used to assess the roll correction feature for clinical feasibility. Applying more stringent pass criteria may yield different results.

In summary, the automatic registration system is capable of accurately detecting induced roll at the level of 0.2 degrees–0.30 degrees, and the automatic roll correction system is capable of accurately correcting for induced roll to within the accuracy of the test described here. After roll correction, all point measurements at the tested angles fall within 2 SD of the baseline average, with the majority falling within 1 SD. Gamma analysis shows that using roll correction brings gamma pass rates to greater than 96% for all measured rotations using 3% difference and 3 mm distance to agreement criteria, and greater than 94% when using 2%/2 mm criteria.

Should a prospective TomoTherapy user wish to incorporate the method described here into routine QA (e.g., monthly QA), we recommend performing this procedure at the time of commissioning and establishing baseline data with a standard DQA plan. Thereafter, one would only need to run the DQA plan twice on a rolled phantom, once with roll correction enabled and once without. This allows a periodic check of roll‐correction functionality while minimally increasing the QA workload.

It should be noted that the location of the treatment couch with respect to the patient changes as the patient rotates in the roll direction. This results in minor deviations in the calculated dose distribution due to the inaccurate representation of the location of the couch with respect to the patient. Such small deviations were not observable in this study.

## IV. CONCLUSIONS

A roll‐sensitive test plan utilizing a common phantom that can be easily reproduced by any TomoTherapy user was designed to perform an end‐to‐end evaluation of TomoTherapy's roll detection and correction capability. TomoTherapy is able to detect rotational misalignments at the level of 0.2°–0.3° using the automatic registration system, and this system showed good agreement with measured induced rotations. Statistical analysis of point data and gamma analyses both show that TomoTherapy's roll correction feature is able to accurately correct for rotational misalignment to within the sensitivity of the test described here. The sensitivity of this test was found to be 0.3° using the ratio of point doses to a normalization point of nonvarying dose. This is the limit at which statistical differences occur between the planned and delivered doses and this level of sensitivity is considered adequate for roll corrections to within clinically relevant tolerances.

While this study provides a technical evaluation of the capabilities of TomoTherapy's roll correction feature, further work is required to evaluate the efficacy of roll correction in clinical circumstances where anatomical deformations accompany rotation.
